# Clinical Outcomes of Early Treatment With Doxycycline for 89 High-Risk COVID-19 Patients in Long-Term Care Facilities in New York

**DOI:** 10.7759/cureus.9658

**Published:** 2020-08-11

**Authors:** Mohammud M Alam, Saborny Mahmud, Mohammad M Rahman, JoAnn Simpson, Sandeep Aggarwal, Ziauddin Ahmed

**Affiliations:** 1 Department of Medicine and Infectious Diseases, Northwell University Hospital, Plainview, USA; 2 Department of Medicine, Johns Hopkins Bloomberg School of Public Health, Baltimore, USA; 3 Department of Medicine, New York University (NYU) School of Medicine, New York, USA; 4 Department of Nursing, Stony Brook University, Stony Brook, USA; 5 Department of Nephrology, University of Pennsylvania, Philadelphia, USA; 6 Department of Medicine and Nephrology, Temple University, Philadelphia, USA

**Keywords:** covid-19, doxycycline, treatment, long-term care facility

## Abstract

Rationale

Due to the cluster and associated comorbidities in residents of long-term care facilities (LTCFs), COVID-19-associated morbidity and mortality are significantly increased. Multiple therapeutic options, including hydroxychloroquine (HCQ) and azithromycin (AZI), were tried initially to treat moderate to severe COVID-19 and high-risk patients in LTCFs, but they were abandoned due to unfavorable reports. As a less toxic option, we initiated treatment with doxycycline (DOXY) very early in the course of illness. DOXY has antiviral, cardioprotective, immunomodulatory, and anti-inflammatory properties, but the efficacy of early intervention with DOXY in high-risk COVID-19 patients in LTCFs is unknown.

Objective

The goal of this retrospective study is to describe the clinical outcomes of high-risk COVID-19 patients with moderate to severe symptoms in LTCFs after early intervention with DOXY.

Design

Case-series analysis

Setting

LTCFs in New York

Participants

This observational study examines 89 patients who were diagnosed with COVID-19 from March 18 to May 13, 2020.

Exposure

All patients who were diagnosed with COVID-19 received DOXY and regular standard of care within 12 hours of the onset of symptoms. Additionally, four patients received meropenem, three patients received Zosyn, two patients received linezolid, and two patients received Bactrim DS. Four patients were on chronic ventilator support. No patients received any steroids or any other antiviral or immunomodulatory agents. The majority of the patients received zinc and calcium supplements as well.

Main outcomes and measures

Assessed measures were patients’ characteristics, fever, shortness of breath (SOB), cough, oxygen saturation/pulse oximetry (POX), radiologic improvements, laboratory tests, DOXY side effects, hospital transfers, and death.

Results

Eighty-nine (89) high-risk patients, who developed a sudden onset of fever, cough, SOB, and hypoxia and were diagnosed with COVID-19, were treated with DOXY (100 mg PO or intravenous (IV) for seven days) and regular standard of care. Eighty-five percent (85%) of patients (n=76) demonstrated clinical recovery that is defined as resolution of fever (average 3.7 days, Coeff = -0.96, p = 0.0001), resolution of SOB (average 4.2 days), and improvement of POX: average 84% before treatment and average 95% after treatment (84.7 ± 7% vs. 95 ± 2.6%, p = 0.0001). Higher pre- and post-treatment POX is associated with lower mortality (oxygen saturation (Spo2) vs. Death, Coeff = -0.01, p = 0.023; post-Spo2 vs. Death, Coeff = -0.05, p = 0.0002). Within 10 days of symptom onset, 3% of patients (n=3) were transferred to hospital due to clinical deterioration and 11% of patients (n=10) died. The result was followed for 30 days from the onset of symptoms in each patient.

Conclusion

Early treatment with DOXY for high-risk patients with moderate to severe COVID-19 infections in non-hospital settings, such as LTCFs, is associated with early clinical recovery, decreased hospitalization, and decreased mortality.

## Introduction

The COVID-19 pandemic placed an unprecedented and overwhelming burden on the U.S. healthcare system. Since the first case of COVID-19 in the U.S., there have been over two million COVID-19 cases and 122,000 deaths as of June 25, 2020 [1]. One-third of these deaths are nursing home residents or workers [2]. Data from small studies conducted in China [3] and France [4] present the clinical outcomes of COVID-19 patients who were treated with hydroxychloroquine (HCQ) and/or azithromycin (AZI). However, since HCQ and AZI are both cardiotoxic, there are concerns about the development of arrhythmia in patients treated with these drugs [5]. With limited treatment options, our group first treated high-risk COVID-19 patients in long-term care facilities (LTCFs) with doxycycline (DOXY) and HCQ [6]. DOXY has anti-inflammatory [7], immunomodulating [8], cardioprotective [9-10], and antiviral [11] activities. Subsequently, other studies noted the harmful side effects and mortality associated with HCQ administration [12].

Based on current evidence and reference articles that detail COVID-19 pathophysiology and DOXY's mechanisms of action, DOXY may be an effective drug in the treatment of COVID-19. As a result, we began treating high-risk patients with only DOXY and supportive care. The efficacy of early DOXY intervention in high-risk COVID-19 patients in LTCFs is unknown. In this observational study, we collected data retrospectively, and we are presenting our clinical observations and outcomes of DOXY for high-risk patients with moderate to severe COVID-19 infections in LTCFs.

## Materials and methods

This case series assessed the clinical outcomes of eighty-nine (89) high-risk patients in LTCFs who developed a sudden onset of fever, cough, shortness of breath (SOB), and hypoxia between March 18 and May 13, 2020. High-risk patients were defined as patients who had at least one comorbidity such as hypertension (HTN), diabetes, coronary artery disease (CAD), congestive heart failure (CHF), chronic obstructive pulmonary disorder (COPD), obesity, or ventilator-dependency. These patients were diagnosed with COVID-19 and they were treated with an early course of DOXY (100 mg PO or IV for seven days) and regular standard of care. Since all patients were closely monitored by healthcare workers, COVID-19 symptoms were immediately noticed; DOXY was started within 12 hours and RT-PCR COVID-19 swab was collected. Assessed measures were patients’ characteristics, fever, SOB, cough, oxygen saturation/pulse oximetry (POX), radiologic improvements, laboratory tests, DOXY side effects, hospital transfers, and death. Clinical recovery was defined as a resolution of fever, resolution of SOB, and improvement of POX (≥93%). Follow-up chest X-rays (CXR) were ordered if clinically indicated.

The statistical analysis was done using Microsoft Excel 2016 (Microsoft Corporation, Redmond, Washington) with statistical application packages and IBM SPSS v23 (IBM Corp., Armonk, NY). The two-tailed T-test was used for between-group intervals in parametric data (e.g. POX). Binary logistic analysis and regression were used to assess the association of risk factors with the outcome. The patients’ outcomes were recorded 30 days after symptoms onset.

All patients or patients’ families gave informed consent before DOXY treatment was started, and oversight medical boards and corporate clinical services approved this observational study.

## Results

Table 1 summarizes the characteristics, clinical features, lab and radiology results, outcomes, and side effects of DOXY of all 89 patients who were started with early DOXY treatment. The median age is 78 years, and the range is 43-101 years. One hundred percent (100%) of patients (n=89) tested positive for COVID-19 through reverse transcription-polymerase chain reaction (RT-PCR) and 85% of patients (n=76) showed clinical recovery. Eleven percent (11%) of patients died (n=10) and 3% of patients (n=3) were transferred to hospitals due to clinical deterioration.

**Table 1:** Characteristics, clinical outcomes, clinical features, lab and radiology results, side effects of doxycycline, and other antibiotics of all 89 high-risk COVID-19 patients who started seven-day doxycycline treatment in long-term care facilities *Clinical recovery defined by resolution of fever (3.7 days), resolution of SOB (4.2 days), and average POX improvement 95% **Four percent (n=4) of ventilator-dependent patients recovered and the ventilator setting returned to baseline. ***Chest X-ray not ordered, not required, or pending. BUN: blood urea nitrogen; CRP: C-reactive protein; LFT: liver function test; LDH: lactate dehydrogenase; POX: pulse oximetry

Assessed Measures	LTCF Residents (n=89)	Percentage
Median Age, Years (Range)	78 (43-101)	
Male	50	56%
Female	39	44%
Clinically Recovered*	76	85%
Transferred to Hospital	3	3%
Death	10	11%
Hypertension (HTN)	62	70%
Diabetes	30	34%
Coronary Artery Disease (CAD)	41	46%
Congestive Heart Failure (CHF)	23	26%
Chronic Obstructive Pulmonary Disease (COPD)	31	35%
Obesity	37	42%
Ventilator Dependency**	4	4%
Fever	87	98%
Shortness of Breath (SOB)	85	96%
Cough	83	93%
Malaise/Weakness	71	80%
Altered Mental Status	56	63%
Diarrhea	31	35%
Chest Pain	27	30%
Tested COVID-19 Positive via RT-PCR	89	100%
High BUN	50	56%
High CR	46	52%
High LFT	33	37%
High Regular CRP	65	73%
High Ferritin	63	71%
High LDH	34	38%
High D-Dimer	57	64%
High Troponin	40	45%
High Procalcitonin	34	38%
Chest X-Ray (CXR) with Pneumonia	85	96%
Chest X-Ray (CXR) Improved	39	44%
Chest X-ray (CXR) Not Improved	41	46%
Chest X-Ray (CXR) Unavailable***	9	10%
Nausea, Vomiting, and Abdominal Discomfort	6	7%
No Side Effects	83	93%
Other Antibiotics	11	12%

Table 2 summarizes the characteristics, clinical features, lab and radiology results, outcomes, and side effects of DOXY of the remaining 76 patients who successfully completed the seven-day DOXY treatment. One hundred percent (100%) of patients demonstrated clinical improvement. After DOXY treatment started, resolution of fever and SOB occurred at an average of 3.7 days and 4.2 days, respectively. Average POX before and after treatment was 84% and 95%, respectively.

**Table 2:** Characteristics, clinical outcomes, clinical features, lab and radiology results, side effects of doxycycline, and other antibiotics of 76 high-risk COVID-19 patients who completed seven-day doxycycline treatment in long-term care facilities This table includes all patients who completed a full course (7 days) of DOXY therapy and excludes any patients who died and who were transferred to hospital and did not complete the seven-day course. *Clinical recovery defined by resolution of fever (3.7 days), resolution of SOB (4.2 days), and average POX improvement 95% after treatment. **Four percent (n=4) ventilator-dependent patients recovered and the ventilator setting returned to baseline. ***Chest X-ray either not ordered, or not required or pending. BUN: blood urea nitrogen; CRP: C-reactive protein; LFT: liver function test; LDH: lactate dehydrogenase; POX: pulse oximetry

Assessed Measures	LTCF Residents (n=76)	Percentage
Median Age, Years (Range)	79 (43-101)	
Male	42	55%
Female	34	45%
Clinically Recovered*	76	100%
Hypertension (HTN)	52	68%
Diabetes	25	33%
Coronary Artery Disease (CAD)	35	46%
Congestive Heart Failure (CHF)	19	25%
Chronic Obstructive Pulmonary Disease (COPD)	26	34%
Obesity	33	43%
Ventilator Dependency**	4	4%
Fever	74	97%
Shortness of Breath (SOB)	72	95%
Cough	71	93%
Chest Pain	21	28%
Malaise/Weakness	62	82%
Altered Mental Status	45	59%
Diarrhea	25	33%
COVID-19 Positive	76	100%
High BUN	39	51%
High CR	39	51%
High LFT	26	34%
High Regular CRP	53	70%
High Ferritin	53	70%
High LDH	27	36%
High D-Dimer	48	63%
High Troponin	32	42%
High Procalcitonin	28	37%
Individuals with Pulse Oximetry (POX) Between 60% and 69% Before Treatment	4	5%
Individuals with Pulse Oximetry (POX) Between 70% and 80% Before Treatment	18	20%
Individuals with Pulse Oximetry (POX) Between 81% and 89% Before Treatment	42	47%
Individuals with Pulse Oximetry (POX) Between 90% and 92% Before Treatment	25	28%
Average Pulse Oximetry (POX) After Treatment	76	95%
Chest X-Ray (CXR) with Pneumonia	72	95%
Chest X-Ray (CXR) Improved	36	47%
Chest X-Ray (CXR) Not Improved	31	41%
Chest X-Ray (CXR) Unavailable***	9	12%
Nausea, Vomiting and Abdominal Discomfort	6	8%
No Side Effects	70	92%
Other Antibiotics	6	8%

There was a statistically significant difference in POX before (pre-POX) and after (post-POX) treatment (n = 89, 84.7 ± 7% vs. 95 ± 2.6%, p = 0.0001). In the multivariate analysis model, the resolution of fever was associated with a reduction in mortality (Coeff = -0.96, p = 0.0001). Higher pre-POX and post-POX were also associated with decreased mortality; lower pre- and post-treatment POX is associated with increased mortality (pre-oxygen saturation (Spo2) vs Death, Coeff = -0.01, p = 0.023; Post-spo2 vs Death, Coeff = -0.05, p = 0.0002). Other variables, such as initial CXR with pneumonia, BUN, creatinine, liver enzyme levels, CRP, ferritin, LDH, D-Dimer, troponin, and procalcitonin, were not associated with mortality. Overall mortality at 30 days in our cohort was 11% (n=10). Clinical improvement was noted in 76 patients. Additionally, 3% (n=3) required transfer to a hospital while the rest of the patients were treated in the nursing home facility.

## Discussion

COVID-19 is a positive-sense, single-stranded ribonucleic acid (RNA) virus [13], with a lipophilic outer shell that allows the pathogen to easily infiltrate lung tissue [14]. The coronavirus is believed to recognize and bind to cluster of differentiation 26 (CD26)/dipeptidyl peptidase-4 (DPP4) markers expressed on the cell surface, serving as an entry point for viral invasion [15]. COVID-19 infects the upper and lower respiratory tract, causing the release of pro-inflammatory cytokines such as interleukin 1β (IL-1β), tumor necrosis factor (TNF), and IL-6 [16]. COVID-19 induces mast cell proliferation within the respiratory submucosa [17] and activates the NF-kB pathway [18], further increasing the inflammatory response. As a result, individuals with COVID-19 present with fever, cough, and SOB. Severe infection can progress to acute respiratory failure and vascular thrombosis. COVID-19 is additionally correlated with acute cardiac injury and increased troponin I levels [19].

DOXY, an analog of tetracycline, is lipophilic and can also easily penetrate the lung epithelium [20]. Chemically modified tetracyclines (CMTs) can also induce apoptosis of mast cells [21]. The drug downregulates inflammatory markers demonstrated to have a role in COVID-19 pathophysiology. Evidence shows that DOXY downregulates the expression of DPP4 through the inhibition of the NF-kB pathway [22], impeding the virus’s ability to enter cells and consequently decreasing the viral load. Along with its anti-inflammatory and immunomodulatory properties, DOXY is considered to be cardioprotective. During reperfusion after myocardial injury, matrix metalloproteinases-2 (MMP-2) are released. DOXY’s inhibition of the MMP-2 pathway rescues left ventricular function [23]. As a result, in patients with acute ST-segment elevation myocardial infarct (STEMI) and left ventricular dysfunction, DOXY reduces adverse left ventricular remodeling [24]. DOXY’s cardioprotective characteristics may improve clinical recovery in COVID-19 patients with acute myocardial injury.

DOXY is also thought to have antiviral activity. In-vitro assays of cultured cells inoculated with the dengue virus suggest that DOXY inhibits the virus’s serine protease, disrupting viral replication, and viral entry into cells [25]. Another study demonstrates that when cells infected with murine retrovirus were treated with DOXY, there was a 70% decrease in retroviral load [26]. Tetracyclines can potentially treat other viral infections as well, such as viral encephalitis, West Nile virus, and human immunodeficiency virus (HIV) [27]. Further research reveals that elevated levels of intracellular zinc inhibit viral replication of COVID-19 [28] and that DOXY facilitates this inhibition by acting as a zinc ionophore, transporting, and increasing intracellular concentrations of zinc. Recently, a study demonstrated that in-vitro, DOXY itself has antiviral activity against COVID-19 at a concentration of 5.6 µM [29].

DOXY has anti-inflammatory, immunomodulatory, cardioprotective, and antiviral properties. No observational study has yet been done using an early regimen of DOXY to treat moderate to severe high-risk COVID-19 patients in an LTCF. This case series demonstrates that early DOXY treatment in a non-hospital environment has good clinical recovery (85%, n=76), minimized hospital transfers (3%, n=3), and reduced death (11%, n=10).

A limit to this study is that there was no control group. We thus compared the data from this population to that of a similar reference patient population in an LTCF in Washington. This study performed an epidemiological investigation regarding 129 confirmed cases of COVID-19. Of the 129 confirmed cases, 63% (n=81) were LTCF residents (median age = 81 years, range = 54-100 years), 26% (n=34) were healthcare workers (median age = 42.5 years, range = 22-79 years, and 11% (n=14) were visitors (median age = 62.5 years, range = 52-88 years). Fifty-six point eight (56.8%) LTCF residents, 35.7% visitors, and 5.9% of healthcare personnel were hospitalized. Comorbidities among LTCF residents included HTN (69.1%), cardiac disease (56.8%), diabetes (37%), obesity (33%), and pulmonary disease (32.1%). Of the total population, 65.1% (n=84) were women. While no deaths occurred among staff members, 27.2% of LTCF residents and 7.1% of visitors died [30]. Comparing this study's data to our data using naive indirect comparison, early DOXY treatment may reduce hospital transfers and decrease mortality.

## Conclusions

In this cohort of high-risk LTCF residents with moderate to severe COVID-19 infections who were treated with an early course of DOXY, improvement in oxygen saturation and resolution of fever were factors associated with lower mortality. The median time of all deaths in these high-risk patients was within the first 10 days in the course of illness. In the majority of our cohort, early initiation of DOXY treatment was associated with improved clinical outcomes, decreased hospitalizations, and decreased mortality. Larger randomized control trials are required to study the effect of DOXY treatment in moderate to severe COVID-19 infections.
